# Supplementary figures and images for: Tumor-Associated Lymphatics Upregulate MHC-II to Suppress Tumor-Infiltrating Lymphocytes
Source: Int J Mol Sci. 2022 Nov 3;23(21):13470. doi: 10.3390/ijms232113470 (PMC9654328; doi:10.3390/ijms232113470)

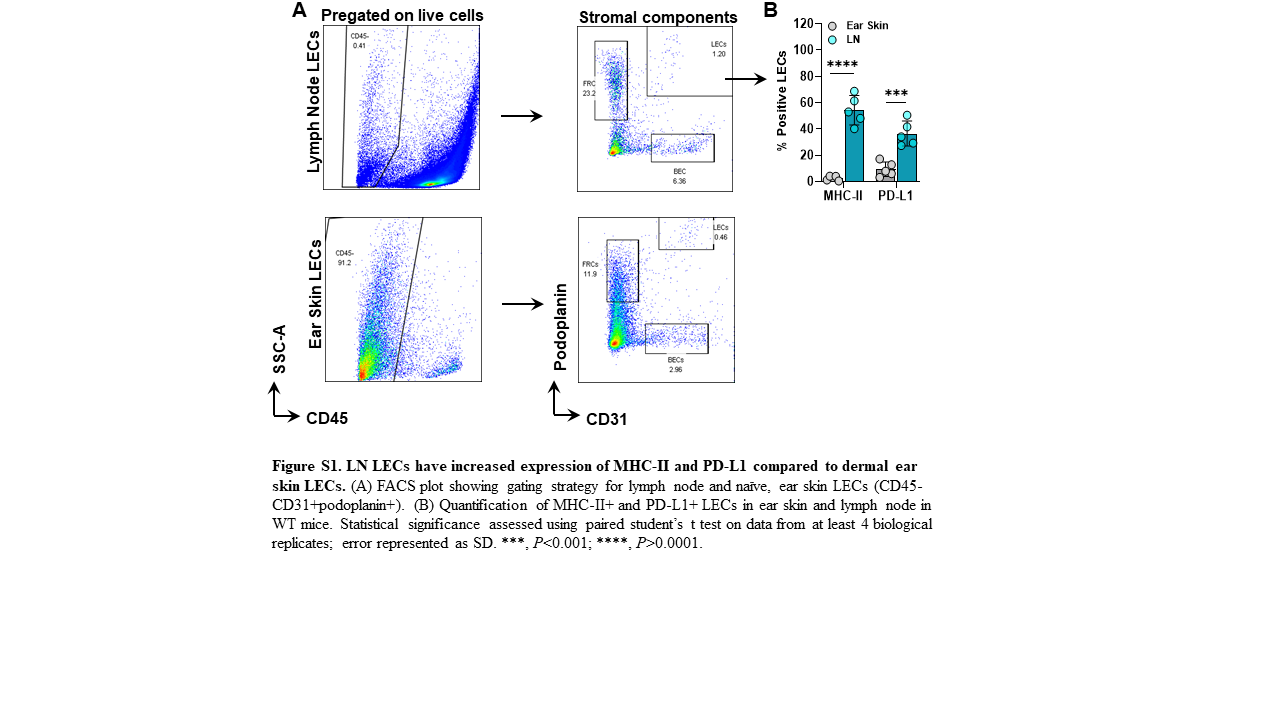

Supplement: Supplementary file 1 [file ijms-23-13470-s001.zip › SuppV5/Figure S1.PNG]

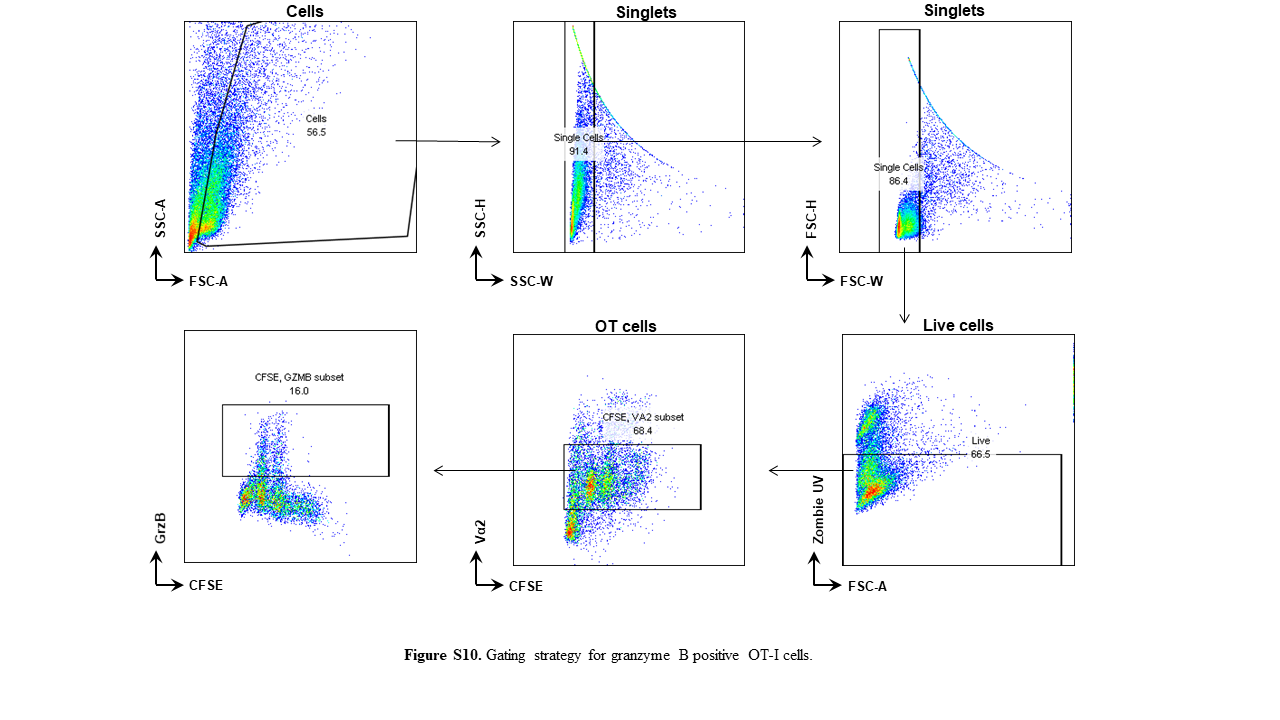

Supplement: Supplementary file 1 [file ijms-23-13470-s001.zip › SuppV5/Figure S10.PNG]

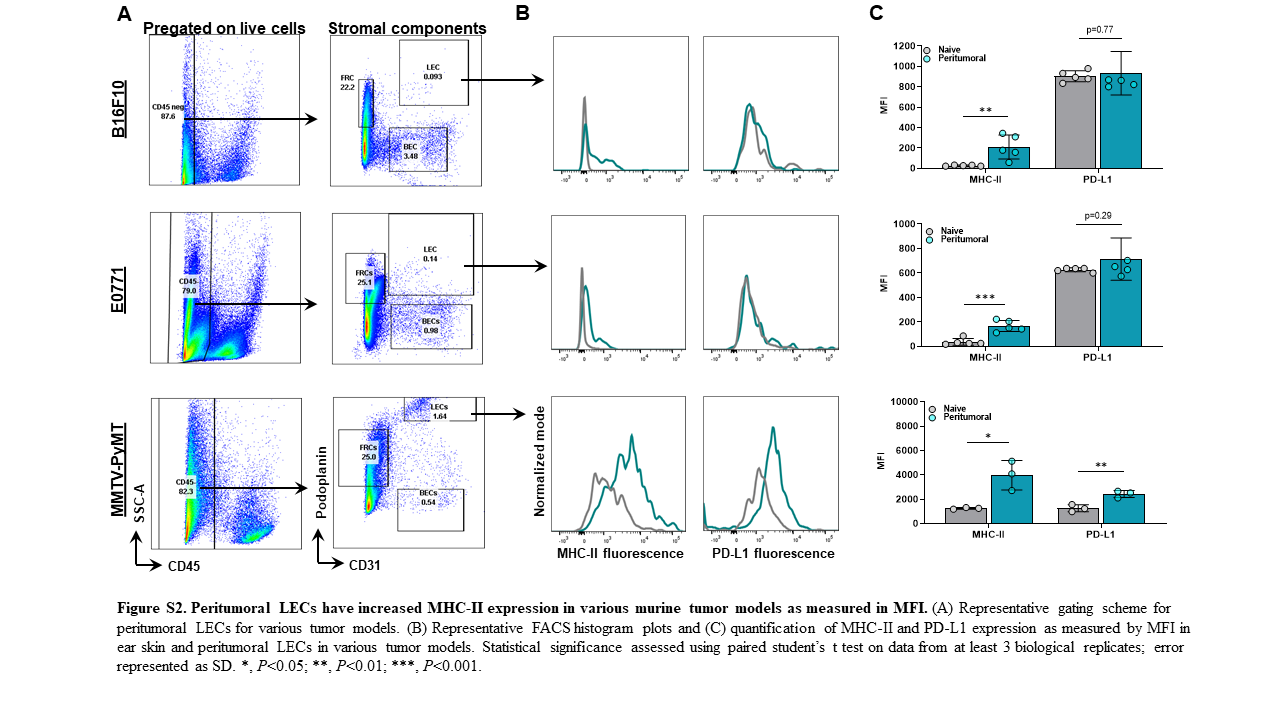

Supplement: Supplementary file 1 [file ijms-23-13470-s001.zip › SuppV5/Figure S2.PNG]

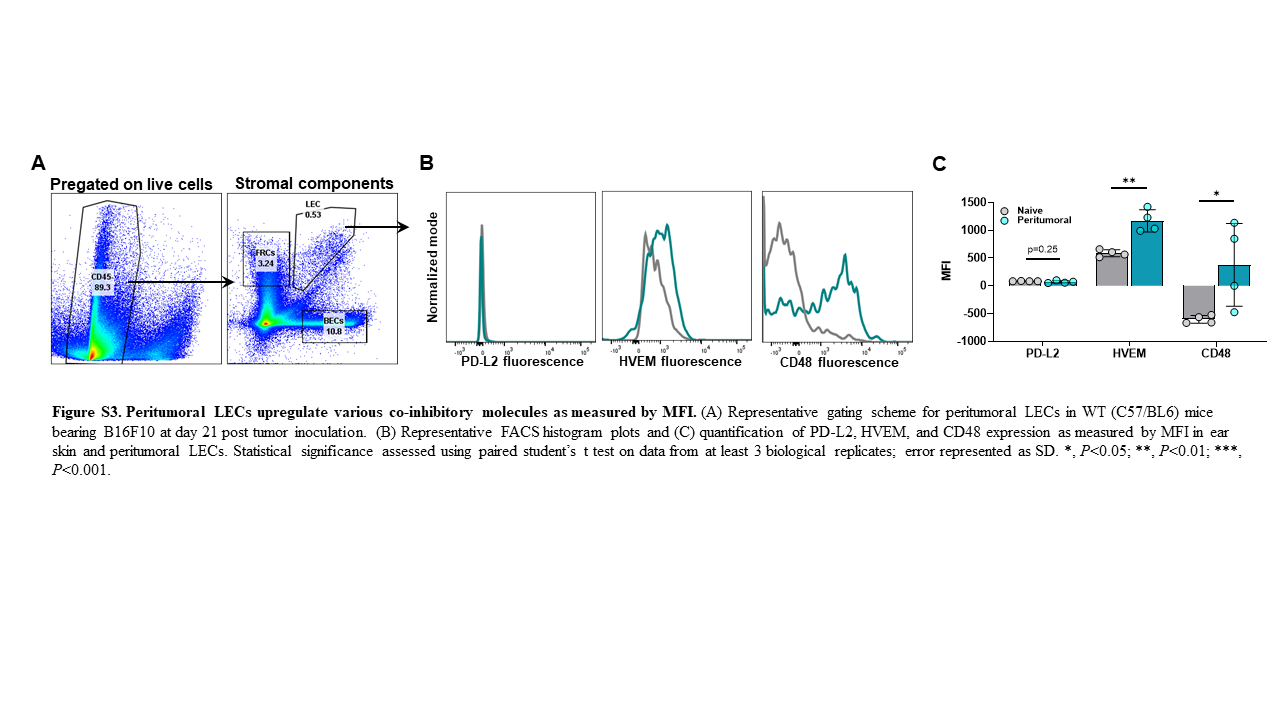

Supplement: Supplementary file 1 [file ijms-23-13470-s001.zip › SuppV5/Figure S3.PNG]

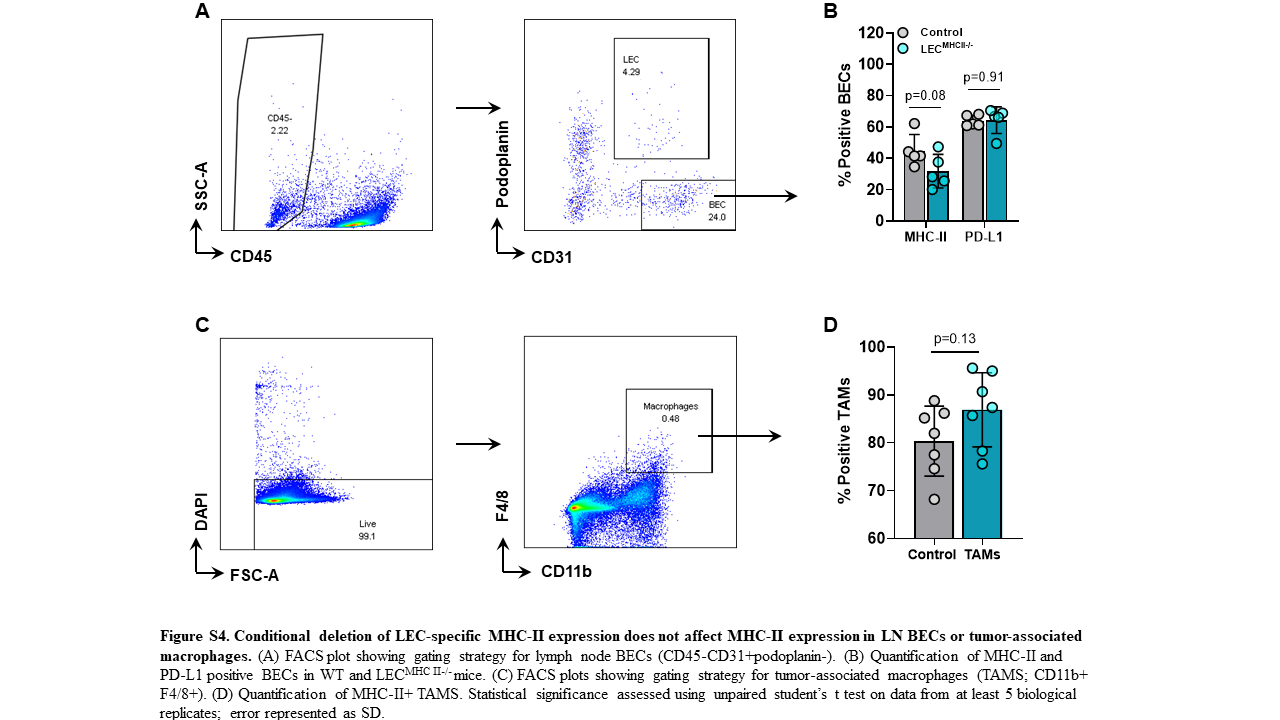

Supplement: Supplementary file 1 [file ijms-23-13470-s001.zip › SuppV5/Figure S4.PNG]

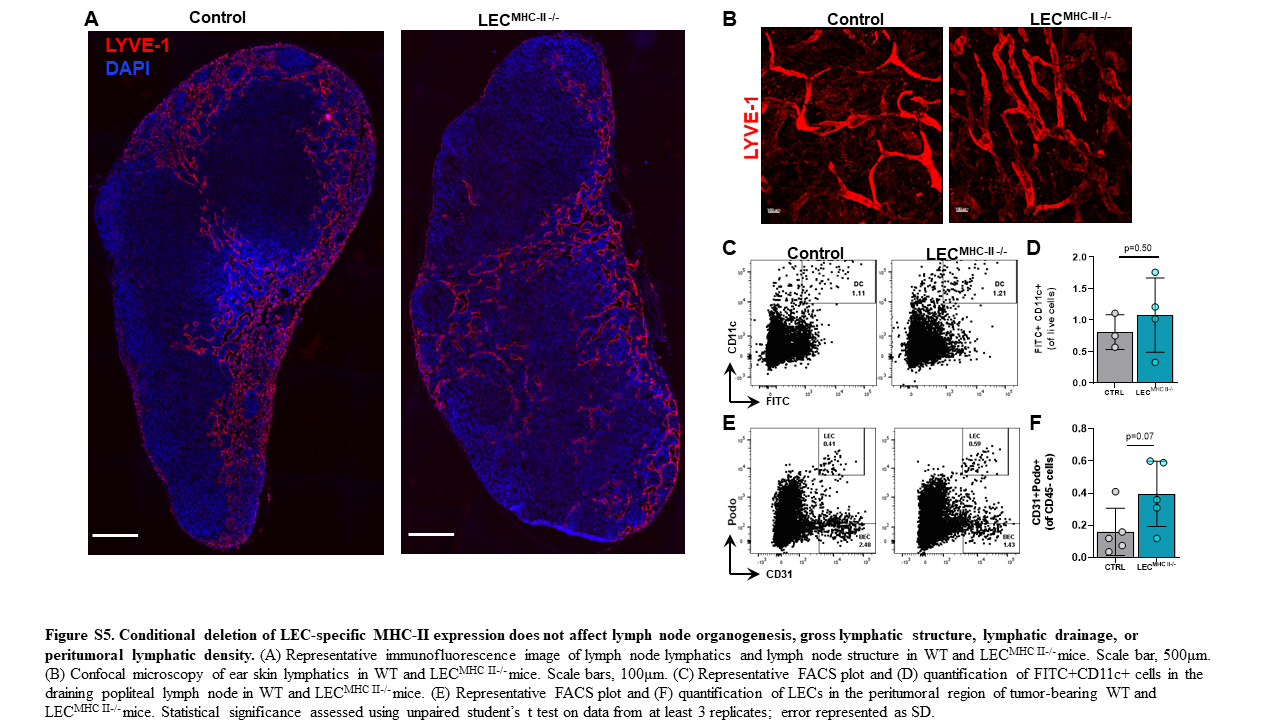

Supplement: Supplementary file 1 [file ijms-23-13470-s001.zip › SuppV5/Figure S5.PNG]

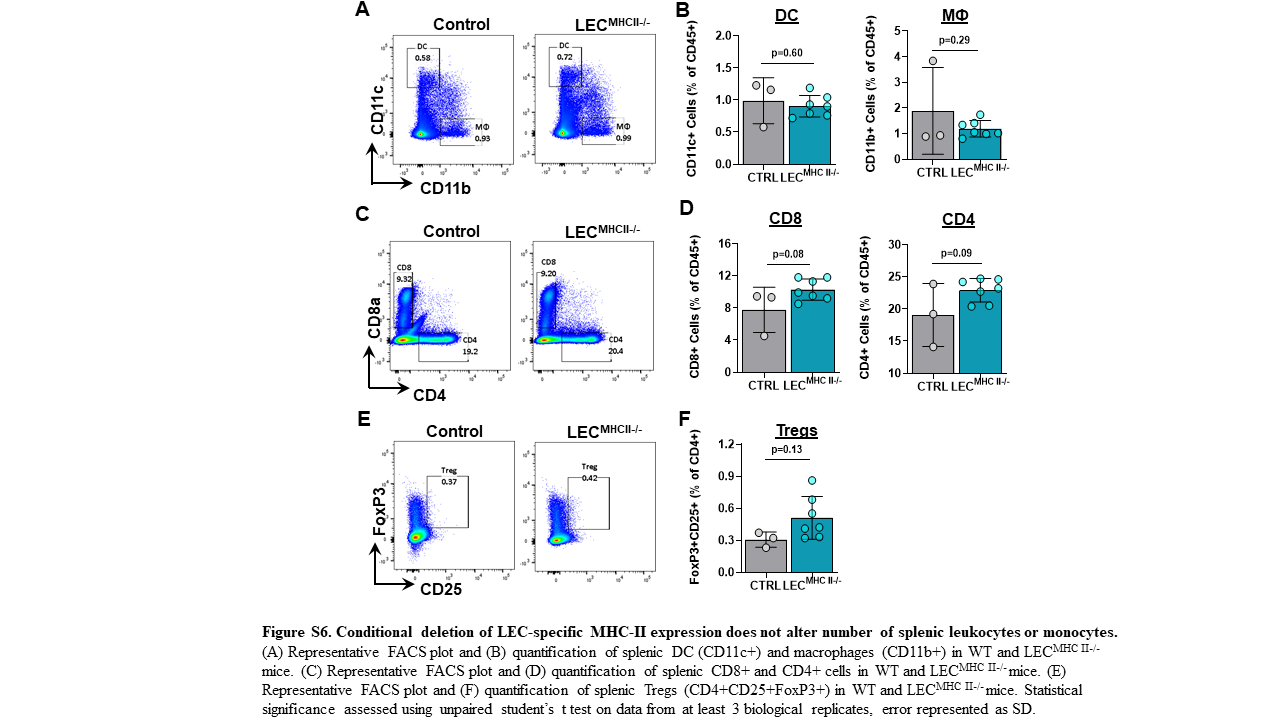

Supplement: Supplementary file 1 [file ijms-23-13470-s001.zip › SuppV5/Figure S6.PNG]

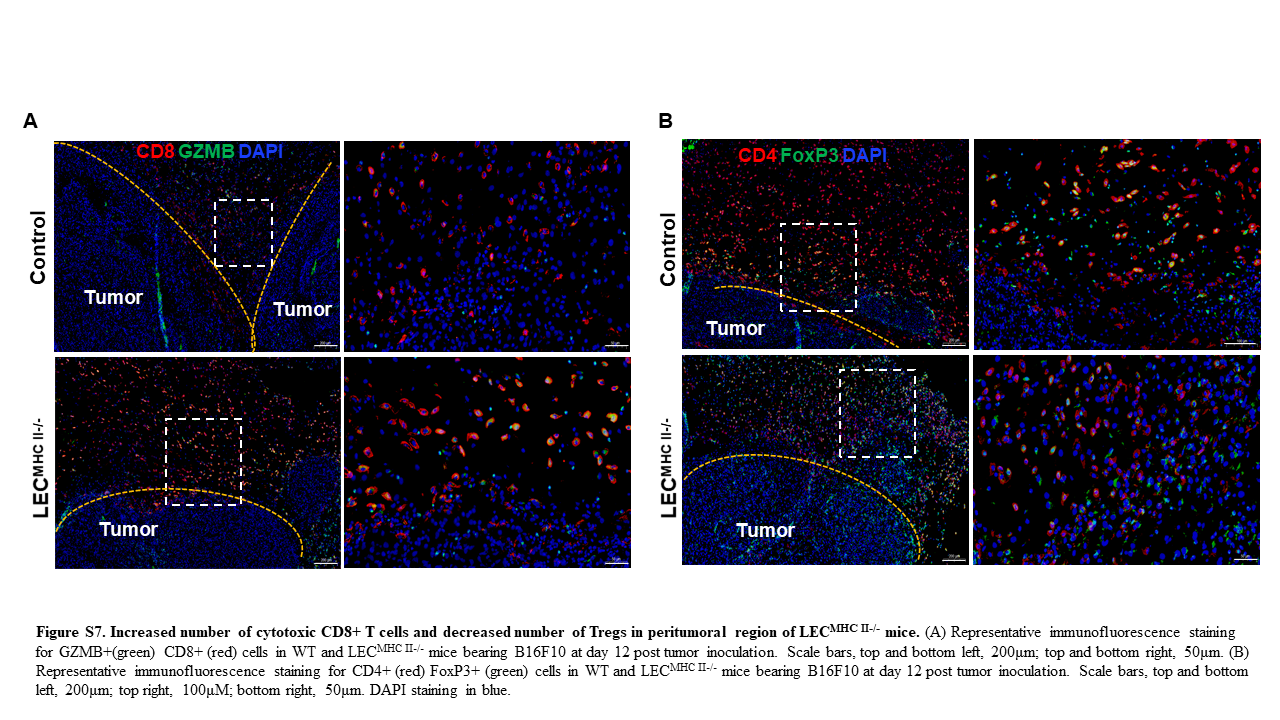

Supplement: Supplementary file 1 [file ijms-23-13470-s001.zip › SuppV5/Figure S7.PNG]

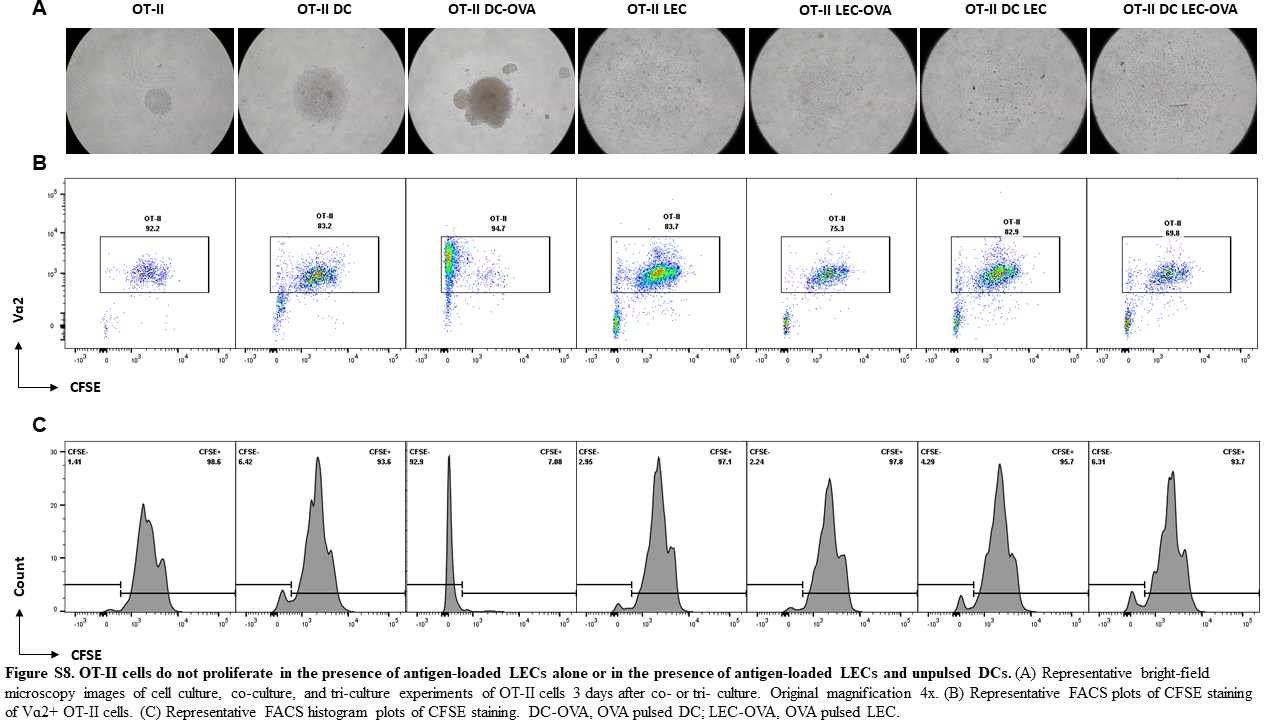

Supplement: Supplementary file 1 [file ijms-23-13470-s001.zip › SuppV5/Figure S8.PNG]

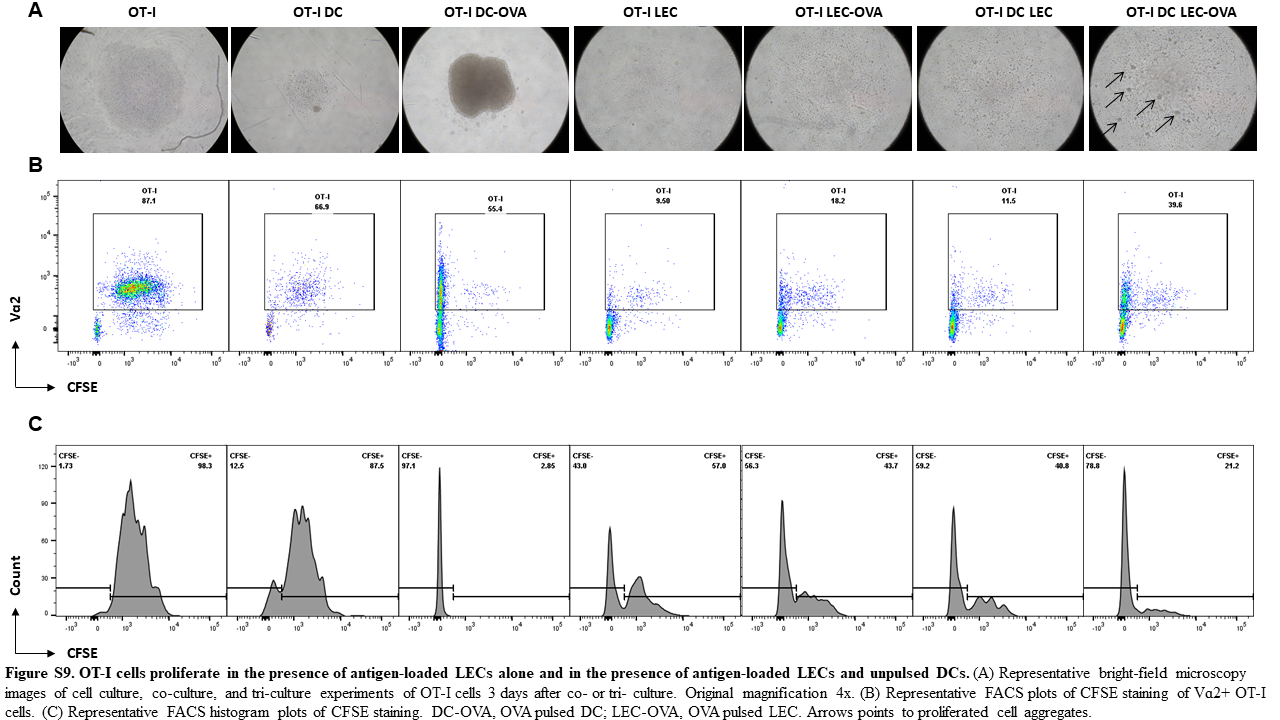

Supplement: Supplementary file 1 [file ijms-23-13470-s001.zip › SuppV5/Figure S9.PNG]

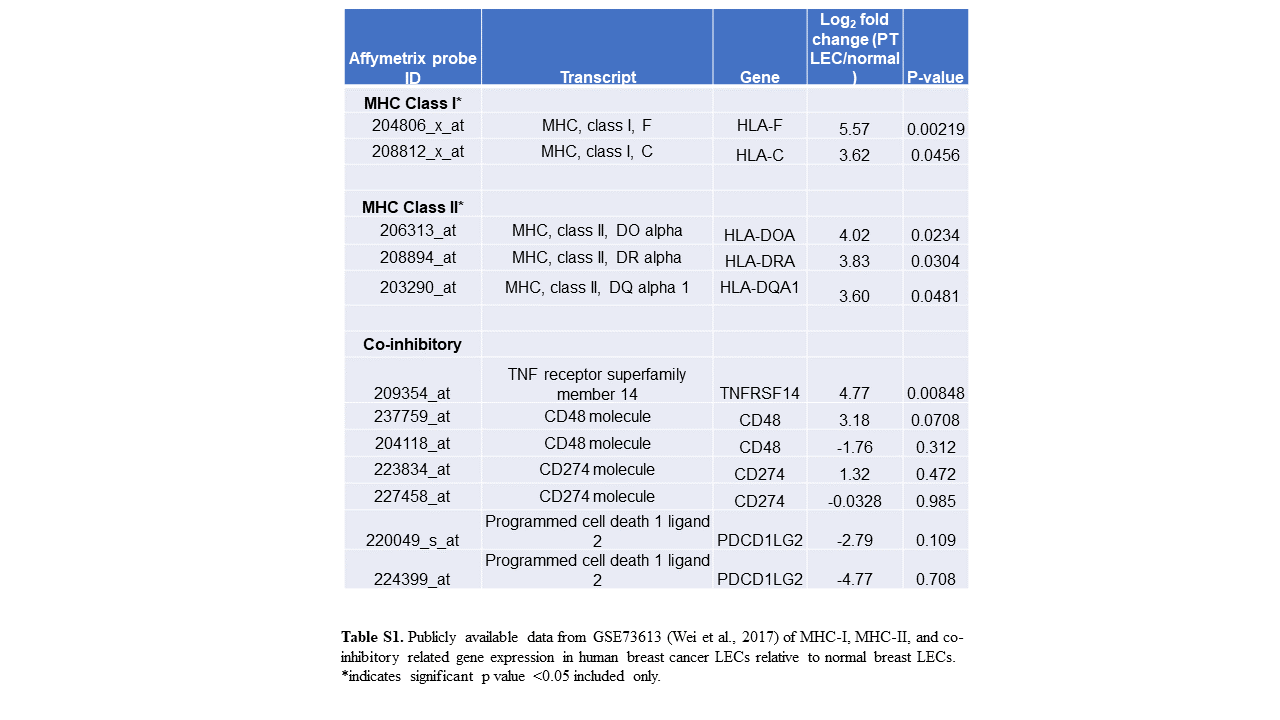

Supplement: Supplementary file 1 [file ijms-23-13470-s001.zip › SuppV5/Table S1.PNG]

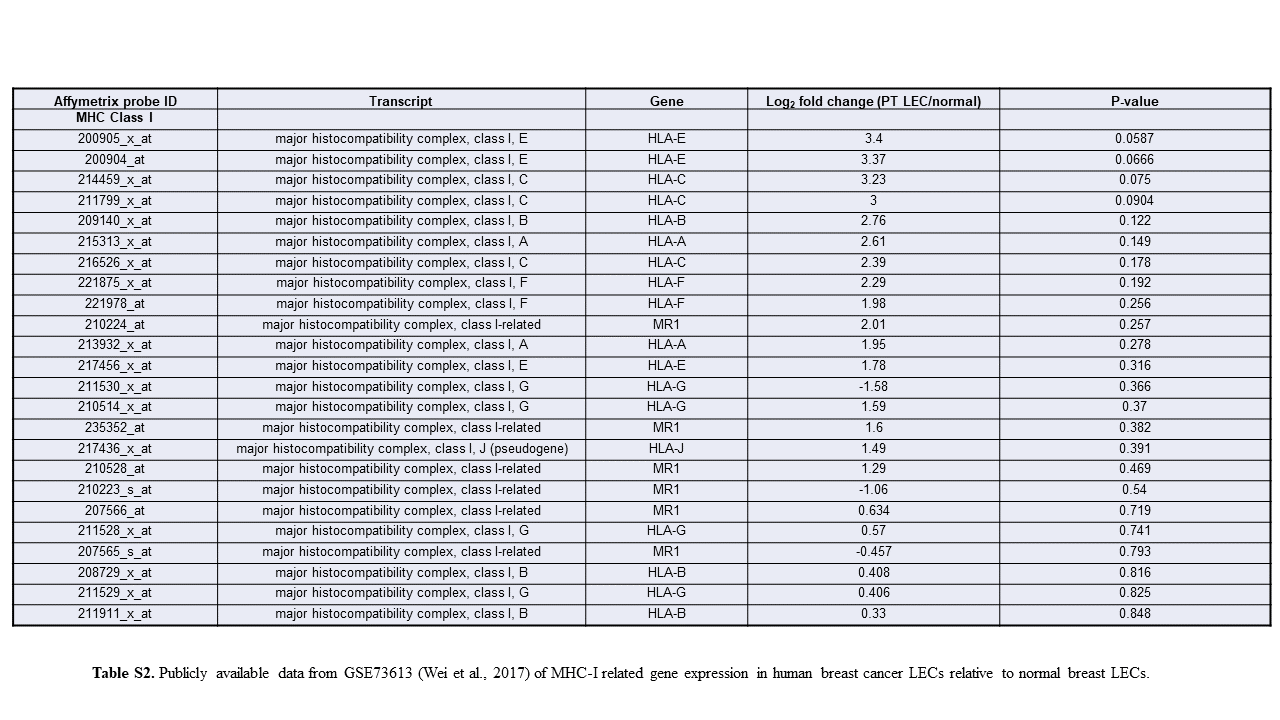

Supplement: Supplementary file 1 [file ijms-23-13470-s001.zip › SuppV5/Table S2.PNG]

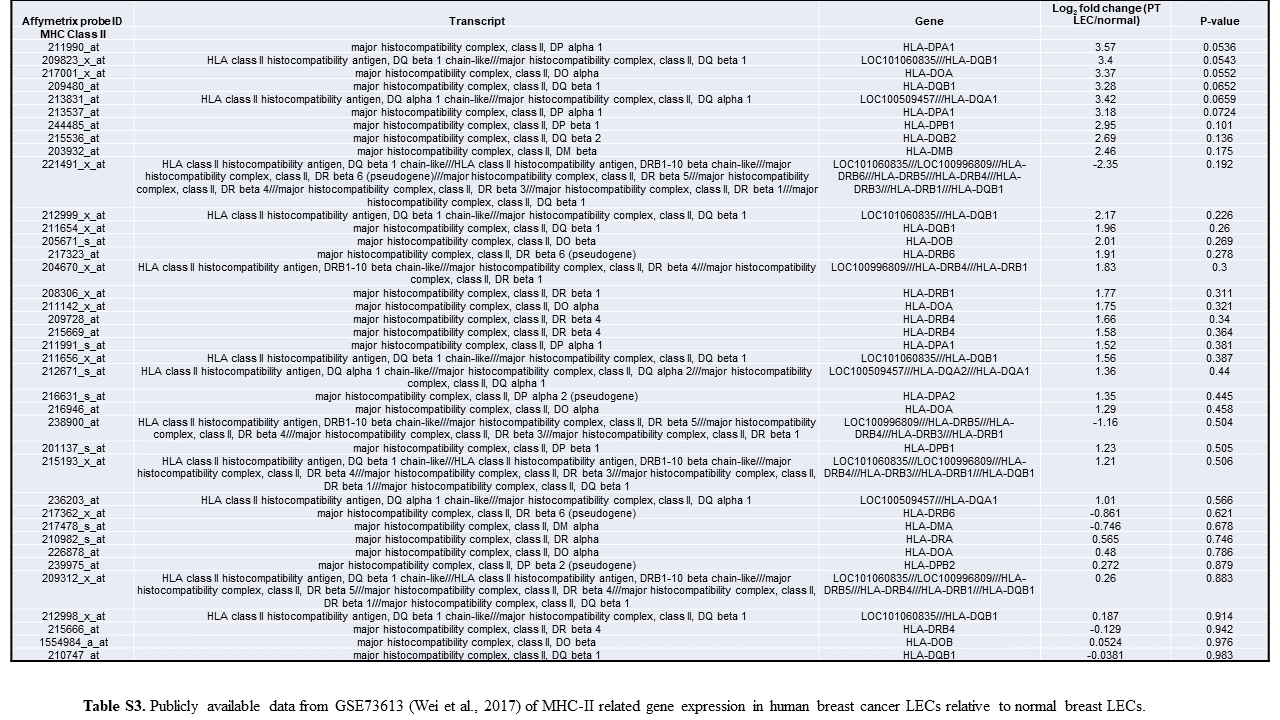

Supplement: Supplementary file 1 [file ijms-23-13470-s001.zip › SuppV5/Table S3.PNG]
